# Supplementary material for: Novel Driver Strength Index highlights important cancer genes in TCGA PanCanAtlas patients
Source: PeerJ. 2022 Aug 11;10:e13860. doi: 10.7717/peerj.13860 (PMC9375969; doi:10.7717/peerj.13860)
Supplement: Supplemental Information 2 [file peerj-10-13860-s002.zip › COHORTS/genes plots/detailed/2021_8_16_14_9_distribution_events_detailed_26.pdf]

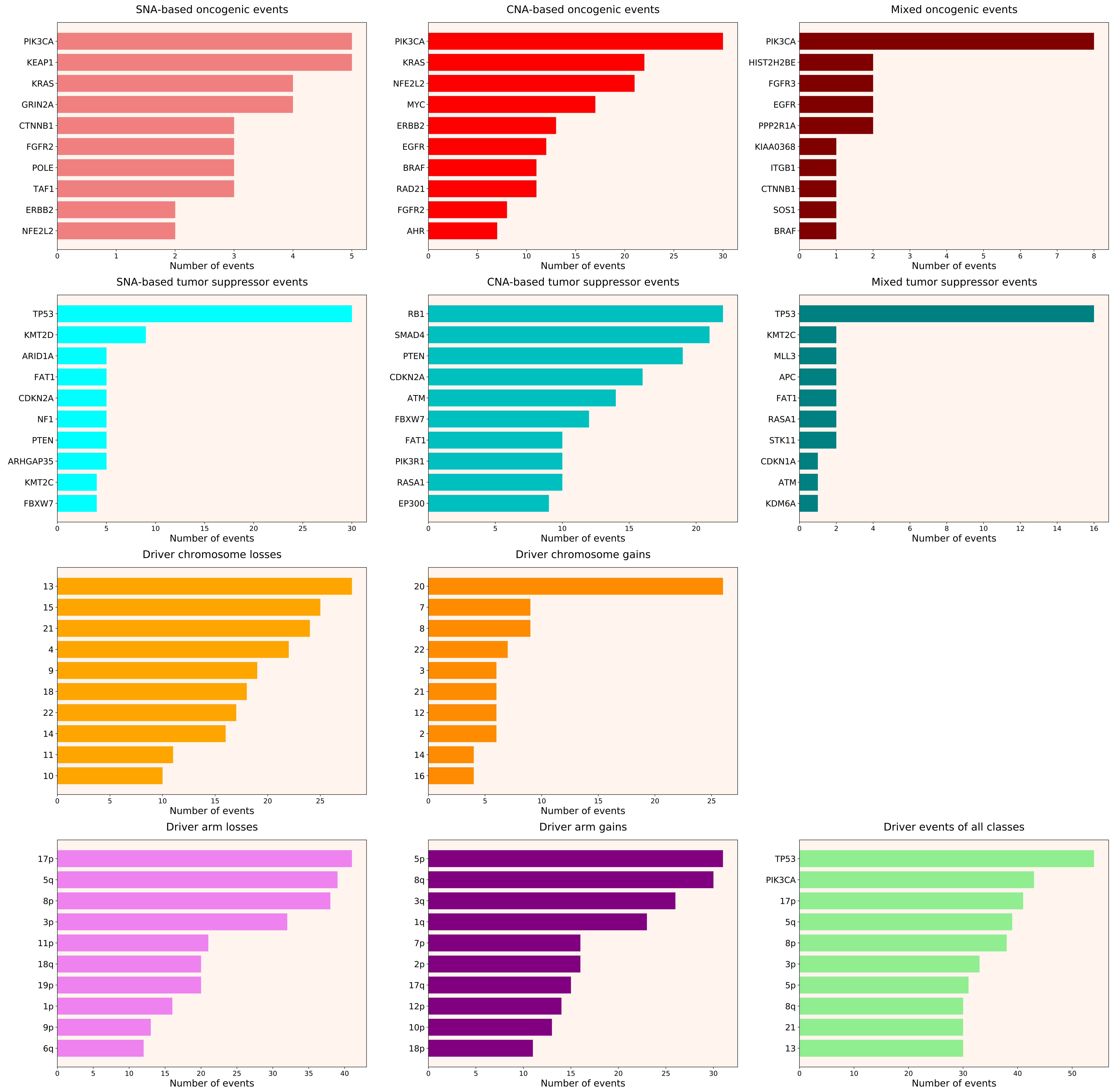

SNA-based oncogenic events
PIK3CA
KEAP1
KRAS
GRIN2A
CTNNB1
FGFR2
POLE
TAF1
ERBB2
NFE2L2
Number of events
CNA-based oncogenic events
PIK3CA
KRAS
NFE2L2
MYC
ERBB2
EGFR
BRAF
RAD21
FGFR2
AHR
Number of events
Mixed oncogenic events
PIK3CA
HIST2H2BE
FGFR3
EGFR
PPP2R1A
KIAA0368
ITGB1
CTNNB1
SOS1
BRAF
Number of events
SNA-based tumor suppressor events
TP53
KMT2D
ARID1A
FAT1
CDKN2A
NF1
PTEN
ARHGAP35
KMT2C
FBXW7
Number of events
CNA-based tumor suppressor events
RB1
SMAD4
PTEN
CDKN2A
ATM
FBXW7
FAT1
PIK3R1
RASA1
EP300
Number of events
Mixed tumor suppressor events
TP53
KMT2C
MLL3
APC
FAT1
RASA1
STK11
CDKN1A
ATM
KDM6A
Number of events
Driver chromosome losses
13
15
21
4
9
18
22
14
11
10
Number of events
Driver chromosome gains
20
7
8
22
3
21
12
2
14
16
Number of events
Driver arm losses
17p
5q
8p
3p
11p
18q
19p
1p
9p
6q
Number of events
Driver arm gains
5p
8q
3q
1q
7p
2p
17q
12p
10p
18p
Number of events
Driver events of all classes
TP53
PIK3CA
17p
5q
8p
3p
5p
8q
21
13
Number of events
